# Sacral Neuromodulation for defecation disorders after non oncologic pelvic surgery

**DOI:** 10.1007/s00384-023-04567-7

**Published:** 2023-12-08

**Authors:** Jacopo Martellucci, Alfredo Annicchiarico, Maximilian Scheiterle, Mario Trompetto, Paolo Prosperi

**Affiliations:** 1 Emergency Surgery, Careggi University Hospital, Florence, Italy; 2 Department of Medicine and Surgery, University of Parma, Parma, Italy; 3 Department of General Surgery, Vaio Hospital, Fidenza, Italy; 4 Department of Colorectal Surgery, S. Rita Clinic, Vercelli, Italy

**Keywords:** Pelvic surgery; Sacral nerve modulation, Colorectal surgery, Obstructed defecation syndrome, Defecation disorders, Constipation

## Abstract

**Purpose:**

Defecation disorders (DD) can sometimes affect the outcomes of pelvic or colorectal surgery. The aim of the present study is to evaluate the role of sacral neuromodulation for the treatment of constipation and other evacuation disorders after surgery.

**Methods:**

A retrospective analysis in all the consecutive patients that underwent sacral nerve modulation (SNM) for DD arisen or worsened after pelvic or colorectal surgery was performed from January 2010 to December 2020. DD were defined starting from Rome IV Criteria, and according to manometric results, all patients were further divided into the two subgroups: inadequate defecatory propulsion and dyssynergic defecation. Cleveland Clinic Constipations Score (CCCS) and SF-36 have been evaluated in the time.

**Results:**

Thirty-seven patients have been included in the study. Twenty-seven out of thirty-seven (73.3%) patients had experienced sufficient benefits to implant the definitive device, and 22 patients (59.4% of tested and 81.5% of permanently implanted) still had the device functioning after a mean follow-up of 6.3 years. The most represented manometric pattern was inadequate propulsive function (59% of patients). CCCS at preoperative assessment for all patients was 17.5 with a reduction to 10.4 at the first year of follow-up (*p* < 0.001).

**Conclusion:**

SNM appears to be a feasible, safe, and well-tolerated procedure with durable benefit in the long-term treatment of defecatory dysfunction after pelvic or colorectal surgery for benign diseases.

## Introduction

It is well known that functional defecation disorders (FDD), characterized by paradoxical contractions or inadequate relaxations of the pelvic floor muscles [1], can result from anxiety, depression, paranoid behavior, obsessions and even sexual abuse [2–4], but little has been known about the onset and behavior of these disorders after surgery. In the analysis of risk factors in the development of benign defecation disorders, surgery, especially rectal surgery, plays a fundamental role [5], but evidence also exist after gynecological surgery or procedures for prolapses, rectoceles. or intussusceptions [6–10].

Moreover, constipation is often preexisting before surgery and may changes its characteristics and severity after surgery.

In fact, chronic constipation (CC) represents a very common gastrointestinal disorder, commonly divided into normal transit constipation (NTC), slow transit constipation (STC), and obstructed defecation syndrome (ODS) [11–16]. However, the definition of CC is not univocal and often differs depending on the point of view of the patient or the clinician. Particularly after surgery, classificatory criteria are even more difficult to use, and symptoms of different functional entities are often associated with each other.

The management of CC is varied and strictly dependent on the etiology of the CC itself with treatments ranging from the simple modification of lifestyles and eating habits [11, 15–18], to use of transanal irrigation (TAI) [16, 19–22] and even surgery [13, 15–17, 23, 24].

Sacral neuromodulation (SNM), at least for part of history, has been part of therapeutic strategies. SNM was first introduced at the University of San Francisco in California by Tanagho and Schmidt, first in dogs and then in humans [25–27]. SNM was initially introduced for urological needs, mainly urinary incontinence due to detrusor instability, and subsequently its benefits were also pointed out on intestinal diseases such as fecal incontinence and CC [28–31]. After an initial enthusiasm, medium-long-term results did not confirm a significant benefit so that, in these years, SNM is no longer commonly used for CC. However, despite the low level of evidence and a recent European consensus statement reports that this procedure is not universally accepted [32], SNM is a minimally invasive procedure and could be considered an alternative to other considerably more invasive surgical procedures [33].

The aim of our study is to evaluate patients with defecatory dysfunction arisen or worsened after surgical procedures, not responsive to behavioral, medical, or rehabilitative treatment, that underwent sacral neuromodulator implantation, in order to establish the long-term efficacy of this method and to identify any categories of patients who would benefit most from this approach.

## Methods

A retrospective analysis in all the consecutive patients with constipation and defecation dysfunctions arisen or significantly worsened after pelvic or colorectal surgery for benign conditions evaluated at the Pelvic Floor Center of the Careggi University Hospital of Florence and at the General Surgery of University Hospital of Siena in the period from January 2010 to December 2020 was performed.

### Inclusion criteria

Patients were included after failure of conventional therapies including laxative use, lifestyle and dietary changes, and pelvic floor rehabilitation. In all the patients, endoscopic and/or radiologic evaluations were performed to demonstrate the absence of significant anatomical alterations or surgical complications conditioning constipation. The defecation disorders were defined starting from Rome IV Criteria [34]. Given the complexity of functional disorders after pelvic surgery, symptoms as clustering or urgency without anal incontinence were also included (Table 1).

**Table 1:** Inclusion criteria for defecation disorders

**In more than 25%% of defecations, two or more of:**
**1. Intensive straining** **2. Lumpy or hard stools (Bristol Scale 1–2)** **3. Sensation of incomplete evacuation** **4. Sensation of anorectal obstruction/blockage** **5. Manual maneuvers to facilitate** **6. Fewer than three evacuation/ week** **7. Need for laxatives or enemas** **8. Clustering** **9. Urgency (without incontinence)**

Although these symptoms are often present simultaneously mixed in various forms, only the one indicated by the patient as most disabling was considered.

Patients with opioid use, irritable bowel syndrome (diagnosed prior to surgery), anal continence dysfunctions (fecal incontinence, gas incontinence, soiling), inflammatory bowel diseases, and neurologic or metabolic primary disease were excluded from the present study.

Patients underwent a proctological evaluation according to the American Society of Colon and Rectal Surgeons guidelines [35]. Patients were asked to complete the Cleveland Clinic Constipation Score (CCCS) [36] and the Italian version of the quality of life (QoL) questionnaire SF-36 [37]. A defecography or MRI to establish anatomical alterations and anorectal manometry to assess sphincter function and rectal sensitivity have been performed. Rectal hyposensitivity was defined as the alteration of at least two values of the three parameters between threshold rectal sensation (TRS), rectal urge sensation (RUS), and the maximum tolerated volume (MTV), compared with the ranges of 50 normal women [38, 39]. A colonoscopy was also performed in all patients with the aim of excluding concomitant organic lesions, and in those with suspected STC, an intestinal transit time was performed according to the Hinton method [40].

Moreover, as required by the Rome IV Criteria for functional defecation disorders [1], according to manometric results, all patients were further studied for inclusion into the two subgroups: inadequate defecatory propulsion (insufficient propulsive forces with or without inappropriate contraction of the anal sphincter) and dyssynergic defecation (inappropriate contraction of the pelvic floor despite adequate propulsive forces).

Post-operative follow-up was performed with clinical evaluation after 30 days from definitive implant, after 12 months, and then annually. During the follow-up period, any additional evaluations required for ineffectiveness or other clinical issues were recorded (i.e., revision of stimulation parameters). During the scheduled follow-up visits, patients were asked to fulfill the CCCS and SF-36 quality of life questionnaire. The efficacy of SNM was evaluated by comparing data at baseline with the data collected at follow-up.

### Surgical Procedure

All surgical operations were conducted by a single operator. The two-stage implantation was performed in the operating room under local anesthesia with basic anesthetic monitoring. After performing local disinfection, a local anesthesia (1% lidocaine and 0.5% levobupivacaine) was performed at the sacral level, around the insertion point of the probe. Both sides are stimulated by a monopolar probe, and the one with the best sensitive (perianal paresthesia) and motor response (anal spastic contractions and ipsilateral big toe flexion) was chosen. Subsequently, under fluoroscopic guidance, a quadripolar probe (Medtronic InterStim^®^ model 3057, Minneapolis, MN, USA) was implanted and connected to a temporary external stimulator (Medtronic InterStim^®^ model 3625, Minneapolis, MN, USA) which was switched on and which the patient kept for about 1 month. The setting parameters of the external stimulator were pulse width of 210 μs, a frequency of 10–30 Hz, and a variable amplitude from 0.1 to 10 V, but they changed during the evaluation period depending on the benefits and from the patient’s sensations. Patients who, after 1 month, had had at least one of the following benefits were considered eligible for definitive implantation: (1) a reduction of at least 50% in episodes of straining, defecatory difficulty, and/or a reduction of at least 50% in episodes of incomplete evacuation; (2) a subjective improvement in symptoms without an increase in the use of laxatives, enemas, or manual stimulation; and (3) an increase in the frequency of bowel movements to more than three per week. All those candidates for the definitive implant underwent a new surgical procedure in the operating room under the same anesthetic and antibiotic prophylaxis conditions as the first procedure. A subcutaneous gluteal pocket was created (generally contralateral to the insertion side of the quadripolar probe but also depending on the patient's morphotype and thickness of the subcutaneous tissue), and the permanent neurostimulator was subsequently positioned (Medtronic InterStim^®^ model 3023 or model 3058, Minneapolis, MN, USA). All patients were re-evaluated 1 week after the procedure for surgical wound assessment.

### Statistical analysis

Statistical analysis was performed using SPSS software (version 16. for Windows; SPSS Inc., Chicago, USA). Results were reported as mean ± standard deviation for continuous variables and number of patients with relative percentage for categorical variables. Comparison between preoperative and follow-up data was made using the *t*-student test; a *p* value < 0.05 was considered statistically significant.

## Results

Between January 2010 and October 2020, 37 consecutive patients evaluated for defecatory dysfunction after pelvic surgery that underwent a SNM first stage implant were included in the study (Table 2).

**Table 2:** Patient’s features

**Variable**		***N* (%)**	**Mean (SD)**
**Demographic characteristics**	**Patients**	37 (100)	
	**Males**	7 (18.9)	
	**Females**	30 (81.1)	
	**Mean age**		53.2 (14.6)
**Manometric pattern**	**Normal pattern**	8 (21.6)	
	**Inadequate defecatory propulsion**	21 (56.8)	
	**Dyssynergic defecation**	8 (21.6)	
	***(Sensitive alterations)***	7 (18.9)	
**Follow-up (years)**			6.3 (3.5)
**CCCS**			17.5 (2.6)

*SD* standard deviation, *CCCS* Cleveland Clinic Constipation Score

The most represented surgical procedures were previous rectopexy or perineal surgery for rectocele or rectal intussusception (STARR, internal Delorme), surgery for endometriosis (III–IV stage), colonic resections (diverticular disease, dolichocolon, volvulus, etc.), or patients that underwent multiple surgical procedures. Also, defecation disturbances after gynecologic pelvic floor surgery were reported. Surgical procedures performed are reported in Table 3.

**Table 3:** Previous surgical procedures

**Type of surgery**	***N* (%)**
Sacral rectopexy	6 (16.2)
Perineal surgery for rectocele or intussusception	6 (16.2)
Endometriosis surgery	6 (16.2)
Colonic resection	5 (13.5)
Hysterectomy and colposuspension	3 (8.1)
Perineal surgery for rectal prolapse	2 (5.5)
Prostatectomy	1 (2.7)
Total colectomy	1 (2.7)
Annessectomy	1 (2.7)
Transurethral prostatic resection	1 (2.7)
Multiple procedures	5 (13.5)

The mean follow-up was 6.3 (SD 3.5) years. Considering manometrical data at baseline, only 8 patients had a normal pattern. An inadequate propulsive function was the most represented (56.8%). After a mean evaluation period of 29.7 days (SD 6.9), an overall number of 27/37 (73.3%) patients had experienced sufficient benefits to implant the definitive device, and 22 patients (59.4% of tested and 81.5% of permanently implanted) still had the device functioning at the last follow-up visit (Table 4). Only one patient removed the temporary device due to infection.

**Table 4:** Main results

**Variable**		**Tested**	**Permanently implanted (% compared to tested)**	**Success at the last follow-up (% compared to tested) [% compared to implanted] **
**Total of patients**		37	27 (73.3)	22 (59.4) [81.5]
**Type of previous surgery**	**Rectopexy**	6	3 (50)	2 (33.3) [66.7]
	**Perineal rectal surgery**	6	5 (83.3)	4 (66.7) [80]
	**Endometriosis surgery**	6	4 (66.6)	4 (66.7) [100]
	**Colonic resection**	5	4 (80)	3 (60) [75]
	**Gynecologic pelvic floor surgery**	3	3 (100)	3 (100) [100]
	**Other**	6	4 (66.6)	3 (50) [75]
	**Multiple procedures**	5	4 (80)	3 (60) [75]
**Manometric pattern**	**Normal pattern**	8	6 (75)	5 (62.5) [83.3]
	**Inadequate defecatory propulsion**	21	17 (80.9)	14 (66.7) [82.3]
	**Dyssynergic defecation**	8	4 (50)	3 (37.5) [75]
	***(Sensitive alterations)***	7	6 (85.7)	6 (85.7) [100]

**Fig. 1:**
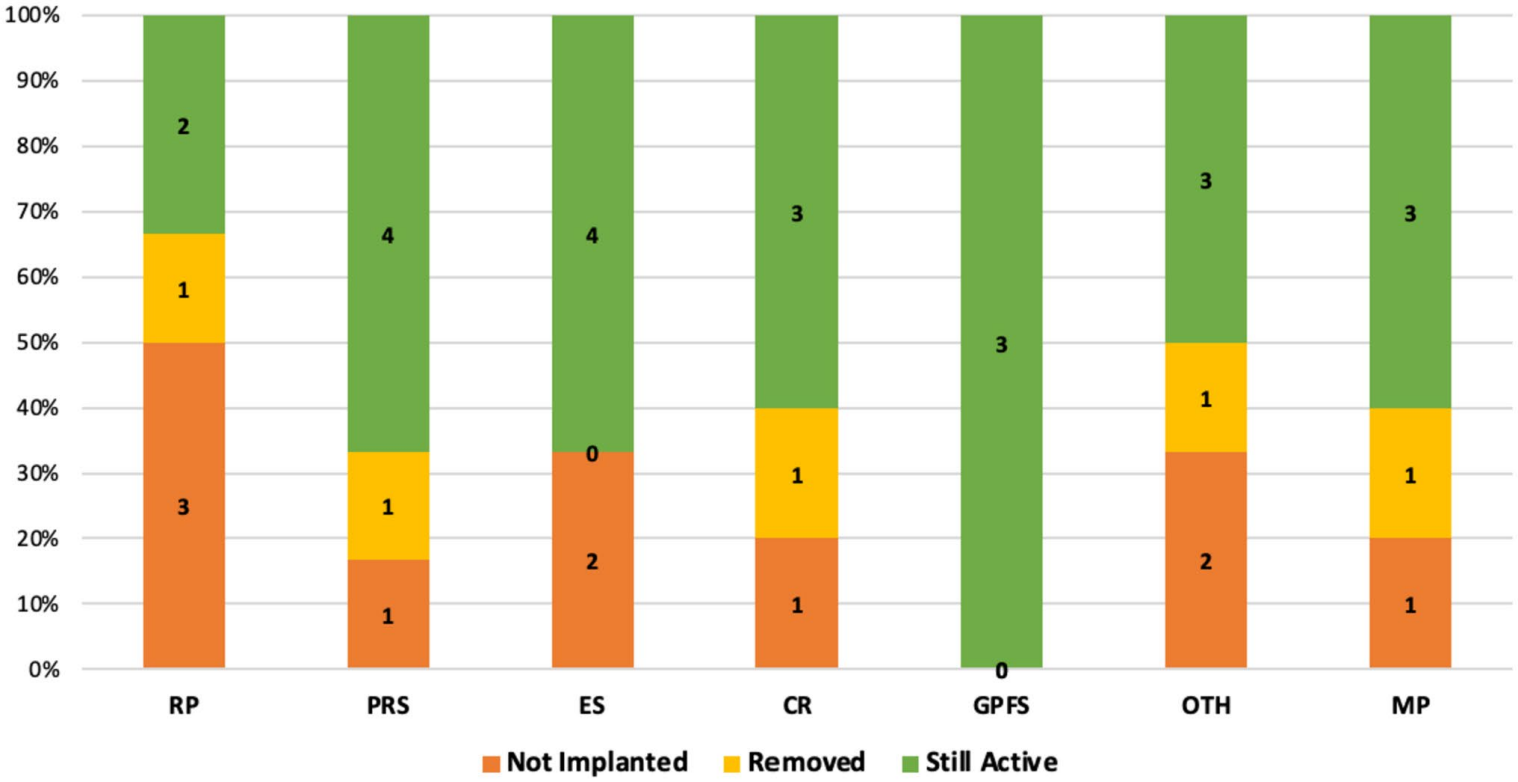
Rate of success. RP, rectopexy; PRS, perineal rectal surgery; ES, endometriosis surgery; CR, colonic resection; GPFS, gynecologic pelvic floor surgery; OTH, other; MP, multiple

Considering patients divided for previous surgery, neuromodulation has proved to be effective in many of the categories, maintaining satisfactory results even in the long term, as shown in Fig. 1. Only in patients treated after rectopexy the implantation rate was about 50%. However, this result was not statistically significant (*p* = 0.3).

Moreover, considering the manometric pattern, even if the success rate of patients with dyssynergic defecation was lower than the other subgroups, this was not statistically significant (p = 0.17).

These results were confirmed at last follow-up, in which the dropout rate was never higher than 25% in any subgroup.

**Fig. 2:**
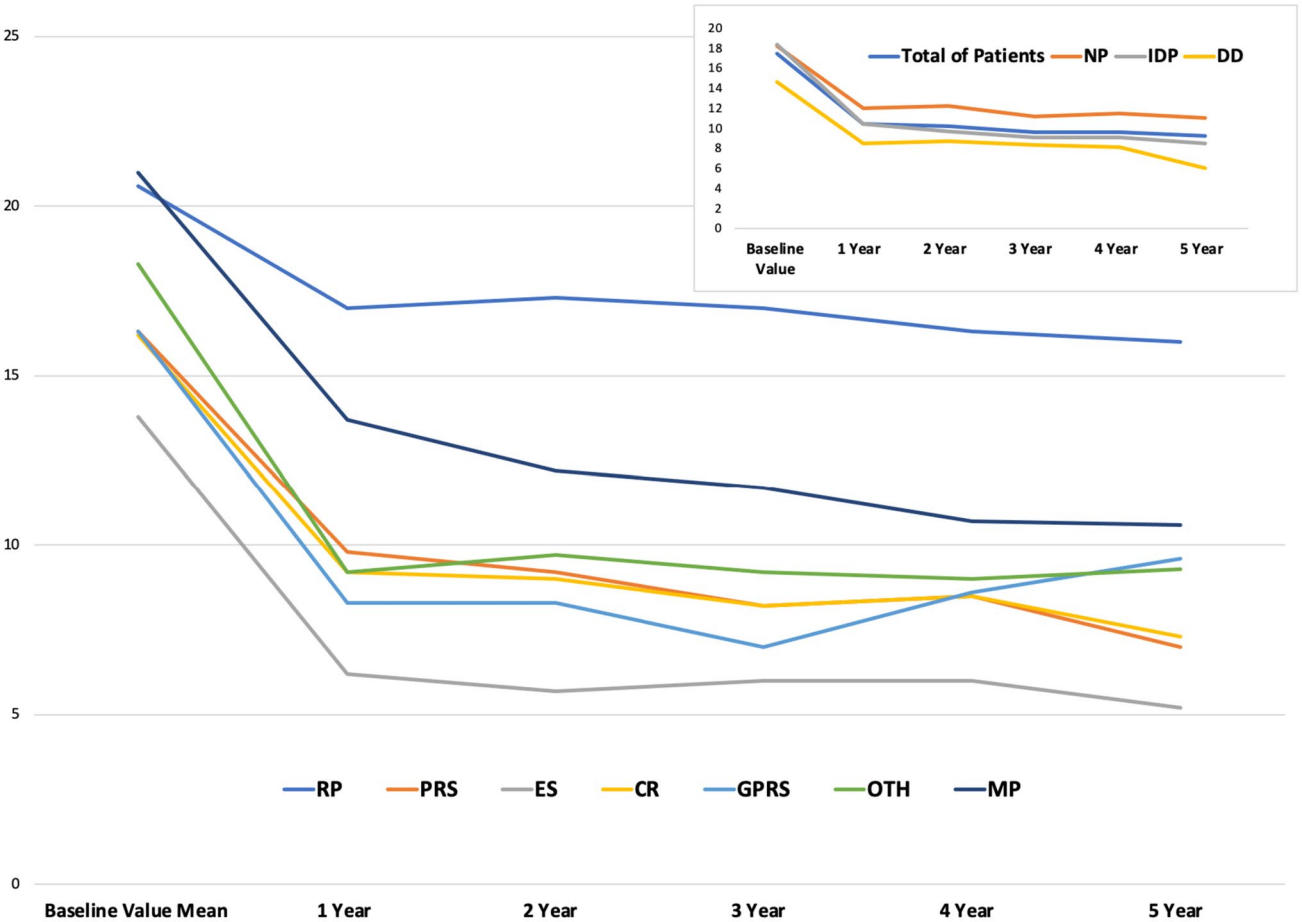
Cleveland Clinic Constipation Score in the time. SD, standard deviation; RP, rectopexy; PRS, perineal rectal surgery; ES, endometriosis surgery; CR, colonic resection; GPFS, gynecologic pelvic floor surgery; OTH, other; MP, multiple; NP, normal pattern; IDP, inadequate defecatory propulsion; DD, dyssynergic defecation

CCCS at preoperative assessment for all patients was 17.5 (SD 2,6) with a reduction to 10,4 (SD 3.6) at the first year of follow-up (*p* < 0.001). This drop was statistically significant even taking into consideration each type of surgery and manometric patterns (Table 5 and Fig. 2).

**Table 5:** Cleveland Clinic Constipation Score in the time

**Variable**		**Baseline value mean (SD)**	**1 year Mean (SD)**	**2 years Mean (SD)**	**3 years Mean (SD)**	**4 years Mean (SD)**	**5 years Mean (SD)**	p*
**Total of patients**		17.5 (2.6)	10.4 (3.6)	10.2 (3.7)	9.6 (3.7)	9.6 (3.2)	9.2 (3.5)	<0.001
**Type of surgery**	**RP**	20.6 (2.1)	17 (1)	17.3 (1.5)	17 (1.7)	16.3 (0.5)	16 (2.8)	0.0295
	**PRS**	16.3 (3.1)	9.8 (1.7)	9.2 (2.7)	8.2 (3.0)	8.5 (1.9)	7 (2.4)	0.0027
	**ES**	13.8 (4.9)	6.2 (1.2)	5.7 (0.9)	6 (1.4)	6 (2.1)	5.2 (2.2)	0.0193
	**CR**	16.2 (3.3)	9.2 (0.9)	9 (2.4)	8.2 (1.2)	8.5 (0.5)	7.3 (1.5)	0.0054
	**GPRS**	16.3 (4.0)	8.3 (1.5)	8.3 (2.5)	7 (2)	8.6 (1.1)	9.6 (4.0)	0.0327
	**OTH**	18.3 (6.6)	9.2 (2.6)	9.7 (1.7)	9.2 (2.3)	9 (4.5)	9.3 (4.0)	0.0332
	**MP**	21 (2.7)	13.7 (2.2)	12.2 (1.7)	11.7 (2.5)	10.7 (2.1)	10.6 (2.5)	0.0037
**Manometric pattern**	**NP**	18.2 (5.3)	12 (4.8)	12.2 (4.6)	11.2 (4.7)	11.5 (4.7)	11 (4.3)	0.0438
	**IDP**	18.4 (3.8)	10.4 (3.4)	9.7 (3.5)	9.1 (3.8)	9.1 (3.3)	8.5 (3.6)	<0.0001
	**DD**	14.6 (4.9)	8.5 (1.8)	8.7 (2.6)	8.3 (2.2)	8.1 (1.9)	6 (1.6)	0.0132

*SD* standard deviation, *RP* rectopexy, *PRS* perineal rectal surgery, *ES* endometriosis surgery, *CR* colonic resection, *GPFS* gynecologic pelvic floor surgery, *OTH* other, *MP* multiple, *NP* normal pattern, *IDP* inadequate defecatory propulsion*, DD* dyssynergic defecation

*Comparison between the preoperative evaluation and the follow-up at the first year

**Fig. 3:**
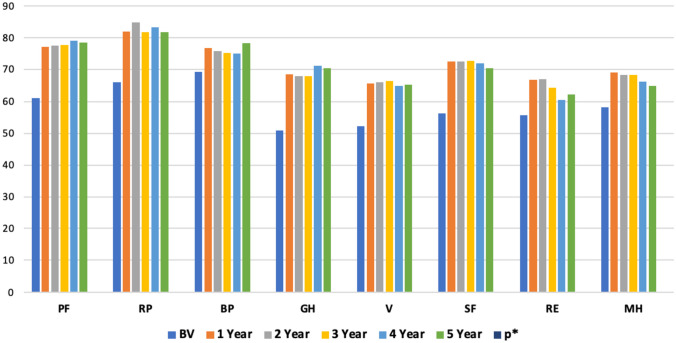
SF-36 evaluation. SF-36, short form health survey 36; BV, baseline values; PF, physical functioning; RP, role physical; BP, bodily pain; GH, general health; V, vitality; SF, social functioning; RE, role emotional; MH, mental health

As reported in Table 6, considering QoL (SF-36), in all categories, the comparison between the preoperative evaluation and the follow-up in the first year was statistically significant (*p*<0.001) with evident improvements on both the physical and psycho-emotional category (Table 6 and Fig. 3). Taking into consideration the individual categories of constipation, however, not all the variables had a statistically significant drop in the comparison of preoperative values with those in the first year of follow-up.

**Table 6:** SF-36 evaluation

**Variable**		**BV**	**FU**	**FU**	**FU**	**FU**	**FU**	p*
			**1 year**	**2 years**	**3 years**	**4 years**	**5 years**	
**Total of patients**	**PF**	61.16	77	77.43	77.7	79.1	78.4	<0.001
	**RP**	65.83	81.87	84.71	81.8	83.3	81.8	<0.001
	**BP**	69.18	76.72	75.7	75.2	75	78.2	<0.001
	**GH**	50.91	68.5	67.9	67.8	71.2	70.4	<0.001
	**V**	52.16	65.75	66.02	66.3	65	65.4	<0.001
	**SF**	56.25	72.5	72.4	72.7	71.8	70.4	<0.001
	**RE**	55.63	66.6	66.97	64.4	60.6	62.2	<0.001
	**MH**	58.13	69	68.2	68.2	66.1	65	<0.001

*SF-36* short form health survey 36*, FU* follow-up, *BV* baseline values*, PF* physical functioning*, RP* role physical*, BP* bodily pain, *GH* general health*, V* vitality*, SF* social functioning*, RE* role emotional, *MH* mental health

*Comparison between the preoperative evaluation and the follow-up at the first year

## Discussion

Literature describes many examples of the onset of constipation after cardiac surgery [41], thoracic surgery [42], bariatric surgery [42], laryngectomies [43], and obviously perineal surgery [6].

SNM for the treatment of constipation has been debated in the past years, with conflicting results. After a significant initial interest, SNM is not currently justified for the treatment of this disorder in many countries. Patton et al., in the long-term analysis of the effects of SNM on 53 patients, showed that only 7% of patients maintained the neurostimulator after a follow-up of 5.7 years [44]. Similarly, Maeda et al. analyzed the data of 62 patients undergoing SNM highlighting how only 14 patients (22%) had maintained the improvements achieved at 60 months and 61% of patients had adverse events related to the implantation of the device [45]. In 2015, a Cochrane review analyzed the effects of SNM in constipation, and although based on only two studies, one of 2 patients and one of 59, respectively, concluded the uselessness of the technique in improving patients’ symptoms [46]. More recently, some randomized clinical trials have confirmed Cochrane’s impressions of the efficacy of SNM on CC. In 2015, Dinning et al. randomized 55 patients with STC undergoing SNM and compared supersensory and subsensory stimulation with sham stimulation without showing any real benefit in the number of weekly bowel movements [47]. Similarly, in another randomized clinical trial, in the 20 of 34 patients who had permanently implanted the device, a positive response was observed respectively in 12/20 and 11/20 after real and sham stimulation (*p* = 0.746). The authors concluded that SNM is not recommended in CC refractory to therapy, even in patients who have responded positively to the evaluation period (PNE) [48].

However, constipation is not a disease, but mainly a symptom that expresses various different primary disorders, often with not effective therapeutic alternatives and deep influence on patients QoL [13, 15, 49, 50].

In contrast to what is already known about the treatment of constipation with SNM, usually oriented on patients with slow transit constipation, promising results were found in the present study on patients with defecation disorders arising or worsened after pelvic or colorectal surgery for benign conditions. In fact, 73% of patients underwent a permanent implant, and 81% of them still have a functioning implant after more than 6 years of follow-up. Considering an intention-to-treat basis, in the medium-long term about 60% of patients still have positive results with this kind of treatment.

This finding can also be considered satisfactory in relation to the few possible therapeutic alternatives, which are often ineffective or even worsening.

In this therapeutical uncertainty, some of the strengths of SNM should be considered the low invasiveness of the procedure, the reversibility, and the relatively low rate of adverse events compared with major surgery performed for refractory constipation [24, 49, 51]. In our study, only 1/37 patients (2%) had to remove the device following early complications after 1 month (infection), and this is also confirmed by other studies [52–54].

The anatomical needs which require surgery could lead to functional alterations at the basis of constipation. Defecatory alterations after surgery can often be related to a dysfunction occurred at the rectal or rectoanal level, although this is often compounded by other complex mechanisms involving bowel transit, rectal (or neorectal) sensitivity, pelvic statics, scar tissue, or other mechanical impairment.

This is the reason why in this study, not only the classic symptoms of constipation were considered but also alterations such as clustering and urgency, which are in any case frequent impairments of the defecatory function in the absence of incontinence.

In this sense, surgical procedures such as correction of abdominal or perineal prolapse, trans-anal rectal resection (STARR), and others could determine conditions of hyposensitivity or impaired rectal propulsion leading to constipation.

Confirming the positive results on these patients demonstrated in our study, rectal hypo-hypersensitivity are known to be associated with fecal incontinence or constipation [55–57] and Knowles in 2012, in a randomized clinical trial of 13 patients, demonstrated that SNS had benefits in those with ODS and rectal hyposensitivity [58].

Considering this, the group of patients with less effective results have been those that underwent abdominal rectopexy. This could be explained by the different potential etiology of the problem, more likely related to mechanical rather than functional alterations. In fact, rectal akinesia was often found after surgical procedures (rectopexy, rectosigmoid resection, etc.), due to the potential limitation of the physiological movements of the rectum during defecation maneuvers [59].

This was also confirmed in ventral mesh rectopexy studies, in which the presence of redundant colon and the pre-existent constipation were associated with an increased risk of persistence of constipation postoperatively or new-onset constipation after surgery [60].

As expressed by the Rome IV Criteria for ODS [1], in our series, we analyzed the potential manometric patterns of dysfunction: inadequate defecatory propulsion and dyssynergic defecation. Although the statistical analysis did not show any differences between the various patterns, our experience seems to suggest a potential predictive role of manometric study.

It is known that endometriosis, especially deep one, can be the cause of chronic pelvic pain and defecation disorders that often force the patients to undergo surgical treatment [61–63]. However, these problems are not always solved by surgery and in some cases may even be worsened. In our experience, 4/6 (66.7%) patients with endometriosis constipation have definitively implanted the neurostimulator, and it seems that all patients have found benefits both in terms of CCCS and QoL even in the long term.

Although this study has some limitations mainly based on the sample size and the retrospective nature of the data, the results reported are encouraging. Patient selection is still mandatory even if, unfortunately, the big question is not so much understanding how and when SNM works, but rather understanding what the pathophysiological mechanisms are underlying constipation in each individual patient [64].

Moreover, although manometric changes are very often present in these patients, the lack of data on manometric studies performed prior to pelvic surgery does not allow us to define an adequate correlation between pelvic surgery, subsequent defecatory disorders, and reported manometric changes. However, this topic appears to be crucial for assessing the real impact of pelvic surgery on the functional outcome and to identify possible predictive factors for success or failure and deserves further research.

## Conclusion

Sacral nerve modulation has shown durable benefit in the long-term treatment of defecatory dysfunction after pelvic or colorectal surgery for benign diseases. SNM appears to be a feasible, safe, and well-tolerated procedure which could offer advantages both in terms of symptomatic improvement and of quality of life. Patient selection remains a key issue to be explored across a larger study population.

## Data Availability

Data archiving is not mandated, but data will be made available on reasonable request.
